# Polyester materials for mRNA delivery

**DOI:** 10.37349/etat.2022.00075

**Published:** 2022-03-11

**Authors:** Wang Chen, Yonghui Ma, Xiaoxuan Liu, Dandan Zhu

**Affiliations:** State Key Laboratory of Natural Medicines and Jiangsu Key Laboratory of Drug Discovery for Metabolic Diseases, Center of Advanced Pharmaceuticals and Biomaterials, China Pharmaceutical University, Nanjing 210009, Jiangsu, China; Donghua University, China

**Keywords:** mRNA, mRNA delivery, polyester, poly(β-amino ester), gene therapy

## Abstract

Messenger RNA (mRNA) has recently made important progress in clinical implementation, offering a promising therapeutic option for infectious disease and cancer. However, the nature of mRNA molecules rendered them poorly bioavailable and unstable *in vivo*, impeding their further clinical application. Therefore, safe and efficient delivery of mRNA therapeutics to the target site is crucial for their successful translation into the clinical setting. Various vectors have been explored for mRNA delivery. Among them, polyesters and their analogs, a family of biodegradable polymers, have exhibited great potential for mRNA delivery. In this short review, the authors briefly introduce mRNA therapeutics, their therapeutic applications and delivery challenges. The authors then presented the typical examples of polyester materials for mRNA delivery to highlight the current progress and discuss the challenges for the rational design of polyester based mRNA delivery vectors. The authors hope to provide a new insight for the design of biodegradable vectors for nucleic acids delivery, thereby promoting their further clinic translation.

## Introduction

**Figure 1.**
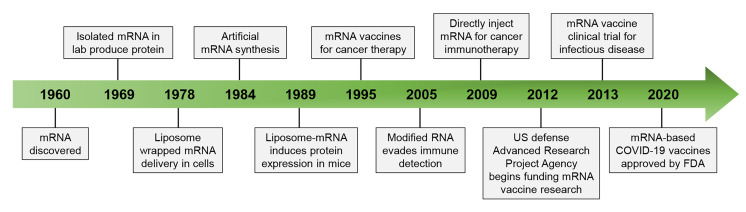
The development history of mRNA technology

Since its discovery in 1960, messenger RNA (mRNA) has attracted increasing attention in both basic and applied research [1, 2]. With the increased understanding of the structure and function of mRNA, mRNA has been emerging as a promising therapeutic modality for the prevention or treatment of diseases (Figure 1) [3, 4]. In particular, two mRNA vaccines that obtained emergency use authorization (EUA) from the US Food and Drug Administration (FDA) in 2020 have exhibited excellent performance in fighting against devastating and evolving coronavirus disease 2019 (COVID-19) pandemic, representing a historic milestone in the therapeutic application of mRNA [5, 6].

However, the clinical implementation of mRNA therapeutics is limited by their poor bioavailability and instability *in vivo* [7–9]*.* To address these obstacles and improve the druggability of mRNA, tremendous efforts have been invested in engineering mRNA molecules and developing mRNA delivery systems [10–13]. Lipids and polymers are the most advanced delivery systems for nucleic acid therapeutics [14–16]. Within the group of polymer vectors, polyester and their analogs hold great potential for mRNA delivery by virtue of their good biocompatibility and biodegradable property.

In this review, we provide a brief overview of mRNA delivery systems, focusing on polyester materials-based mRNA delivery systems. We started with a short introduction to mRNA therapeutics and their delivery challenges.

## mRNA therapeutics and their delivery challenges

Based on the central dogma, mRNA is the bridge that links and directs genetic information flow from DNA to proteins. Once introduced into cells, mRNA can be translated to the corresponding protein via the protein synthesis machinery. In principle, mRNA is able to regulate the function of any gene in living organisms. Accordingly, mRNA-based therapeutics have been exploited in various biological and medicinal applications, including protein replacement therapy, vaccines for infectious diseases and cancer, genome editing, cell reprogramming, etc [17–22].

**Figure 2.**
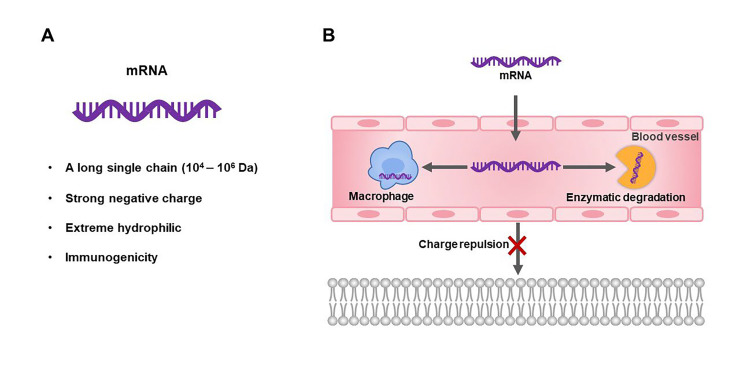
Structural properties and delivery challenges of mRNA therapeutics. A. The structural characteristics of mRNA; B. the schematic diagram of the intracellular and extracellular obstacles that mRNA therapy needs to overcome

Despite the promising of mRNA therapeutics, they are, unfortunately, not favorable drug molecules. mRNA molecules have a large molecular weight (10^4^–10^6^ Da) and high negative charges, which lead to negligible cellular uptake. And, mRNA molecules are easily degraded by 5’ exonucleases, 3’ exonucleases, and endonucleases. They also are recognized and clear by macrophages [23] (Figure 2). Another important issue of mRNA is its immunogenicity. Exogenous mRNA can be recognized as the viral infection signal to trigger innate immunity, such as the pathogen-associated molecular pattern (PAMP) recognized by the retinoic acid-inducible gene I (RIG-I) [24–26]. Therefore, the development of advanced delivery vehicles that could protect mRNA from degradation and deliver them to the site of action is pivotal for the biomedical applications of mRNA therapeutics.

**Figure 3.**
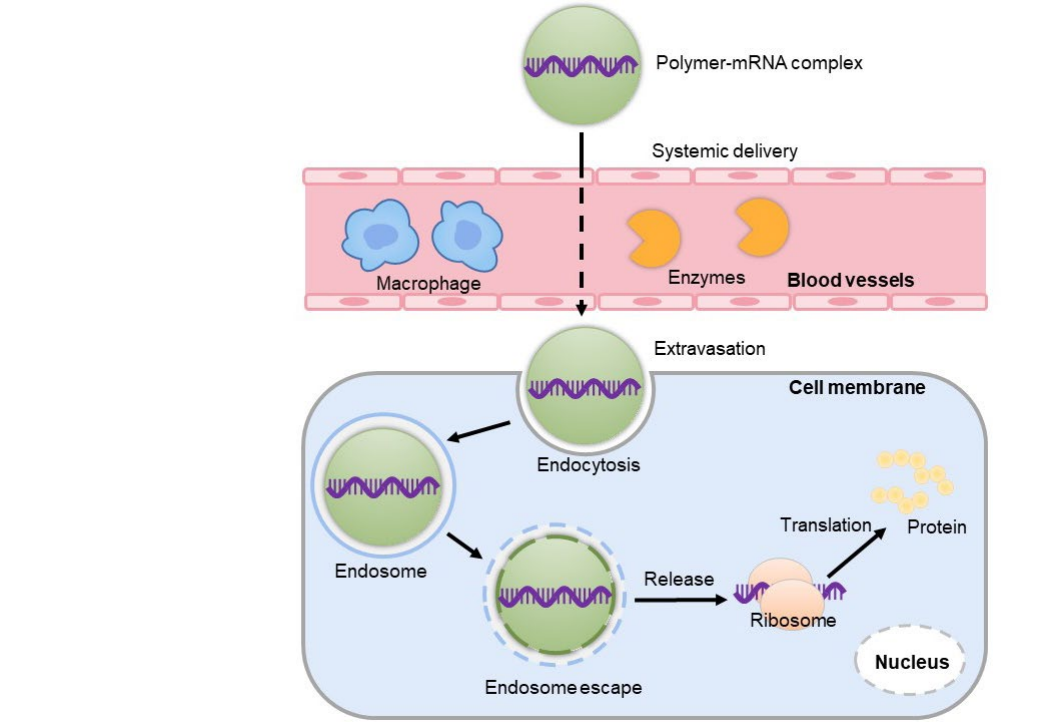
Polymer vectors mediated mRNA delivery. Polymers can encapsulate mRNA and overcome many obstacles to promote the uptake by target cells and then must be released into the cytoplasm to successfully express the protein

Both viral and non-viral vectors have been developed for mRNA delivery. Despite the advantages of viral vectors in terms of delivery efficacy, their clinical application is hampered by the high risk of immunogenicity, high production cost, and biosafety issues. Researchers, therefore, shifted their interests to develop non-viral delivery vectors for mRNA therapeutics by virtue of their good compatibility, low risk of immunogenicity, and ease of production [27]. In the past decades, various delivery materials have been invented for the safe and efficient delivery of mRNA therapeutics [23, 28, 29]. Among them, polymer-based systems are the most advanced vectors for nucleic acid therapeutics. For instance, a cyclodextrin-based polymeric vector has been employed in the first clinical trial of small interfering RNA (siRNA) therapeutics [30], lipid-based nanoparticles (LNPs) have been recently employed as delivery vehicles for COVID-19 mRNA vaccines by Pfizer/BioNTech and Moderna for emergency application [12, 31]. Although currently less advanced than LNPs in the clinics, polymeric vectors are able to meet the *in vitro* and *in vivo* delivery challenges of mRNA therapeutics by virtue of their versatile functional modification, holding great potential for mRNA delivery. Polymeric vectors can stably encapsulate mRNA, efficiently deliver mRNA to the target site, promote the uptake of target cells, facilitate effective endosome escape and release mRNA to initiate translation of functional proteins (Figure 3) [11].

## Polyester materials for mRNA delivery

**Figure 4.**
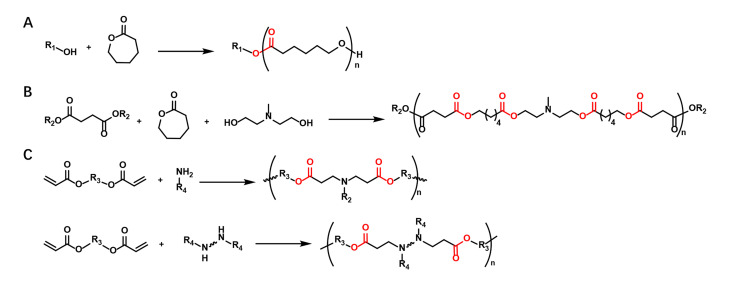
Synthesis of the ester-containing polymers via either ROP (A and B) or stepwise-growth polymerization, e.g., Michael addition reaction (C)

Various types of polymers have been explored for mRNA delivery. Among them, biodegradable polymers containing ester linkages have shown great potential in this field because of their biocompatibility, flexibility and efficiency [32–35]. The ester-containing polymers employed for mRNA include polyester, poly(β-amino ester) (PBAE), poly(amine-co-ester) (PACE), poly(2-(dimethylamino)ethyl methacrylate) (PDMAEMA), poly(lactic-co-glycolic acid) (PLGA), poly(lactic acid) (PLA), etc. They are generally prepared by either ring-opening polymerization (ROP), or stepwise-growth polymerization, e.g., Michael addition reaction (Figure 4). Both strategies allow easy synthesis of polymers via either one or two steps and the polymer size can be controlled by modulating the stoichiometry. The ROP reaction can be prepared by two different mechanisms: 1) step-growth polymerization or condensation; 2) ring-opening polyaddition (chain polymerization). The step-growth polymerization relies on hydroxy acid condensation or condensation of a mixture of a diacid and a diol. However, the condensation reaction usually requires a high temperature and long reaction time, which is prone to side reactions. The polymerization of lactams and lactones by ring-opening addition can overcome these limitations. Functional polyesters of high molecular weight can be easily prepared from lactones of different ring diameters under mild conditions [36]. The stepwise-growth polymerization is typically employed to construct PBAE based materials. For instance, linear PBAEs are prepared by conjugating the bis(acrylate ester) backbone monomer with primary or secondary amines of the side chain monomer(s) via Michael addition reaction [34]. We will showcase the latest developments in the use of polyester-based nanoparticles for mRNA delivery in this section.

**Figure 5.**
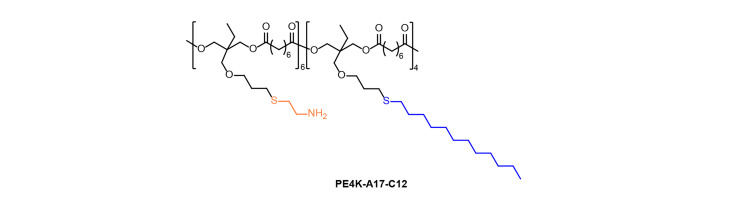
Chemical structure of polyester PE4K-A17-C12

Due to the unique physical and chemical properties of single-stranded mRNA, researchers should fully consider the balance of degradability and functionality, as well as the requirements for safety, stability, and transfection efficiency when designing functional polyester carriers for mRNA delivery [37, 38]. For example, Yan et al. [39] developed a robust functional polyester library for mRNA delivery using the polycondensation of trimethylpropyl allyl ether and diacyl chlorides. The candidate A17 (cysteamine)—modified polyesters series were identified and validated for luciferase encoding mRNA (Luc mRNA) in ovarian cancer IGROV1 cells. Especially, the candidate polyesters (PE4K-A17-C12) (Figure 5) were demonstrated selective mRNA delivery into the lung after formulation with 5% Pluronic F127 after intravenous injection into mice, due to the 5% F127 formulated PE4K-A17-C12/mRNA complexes have a better balance of the protective effects and delivery potency for mRNA delivery. This study highlights the optional balance of stability and delivery potency for polyester-based mRNA formulation [39].

**Figure 6.**
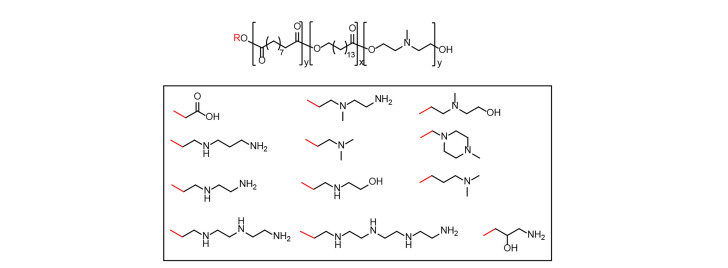
Representative chemical structure of PACE

Noteworthy, to challenge the “bottom-up” construction of polymer libraries, Jiang et al. [32] developed a “top-down” process to establish biodegradable PACE terpolymer for mRNA delivery (Figure 6). The PACE terpolymers contain three key features: hydrophobic lactone monomers for the stability of the formulation, ester linkage in the main chain for biodegradability and tertiary amine with low cationic charge density for electrostatic interaction with nucleic acids and avoiding toxicity. Several actuated PACE (aPACE) materials (Figure 6) exhibited superior mRNA delivery properties *in vitro*, compared with regular PACE. Importantly, aPACE could successfully deliver the erythropoietin (EPO) mRNA *in vivo* and enhance the expression of EPO with negligible systemic toxicity. This new and safe aPACE provides a new perspective for developing the biodegradable mRNA delivery vehicle [32].

Furthermore, to investigate the relationship between the endosomal escape ability and transfection efficiency of poly(amino ester) based mRNA nanoparticles, Jiang et al. [33] recently firstly used a deglycosylation-dependent Renilla luciferase derived (ddRLuc) molecular probe to quantify endosomal escape and mRNA transfection efficiency of PACE polymers mediated mRNA delivery system. The activity of a novel luciferase probe can be restored only in the cytosol, which reliably and sensitively monitors intracellular traffic of poly(amino ester) based mRNA formulation. Those PACEs with different terminal groups could be co-encapsulated with firefly luciferase (FLuc) mRNA and ddRLuc to observe a strong correlation between endosomal escape and transfection efficiency. The preferred PACE could specifically deliver mRNA into the spleen or lymph nodes. This study highlights that mRNA encapsulation efficiency and endosomal escape, but not cellular uptake, were determinant factors for transfection efficiency for intracellular poly(amino ester) based mRNA delivery systems. Also, these PACE had great potential for mRNA-based vaccine delivery [33].

**Figure 7.**
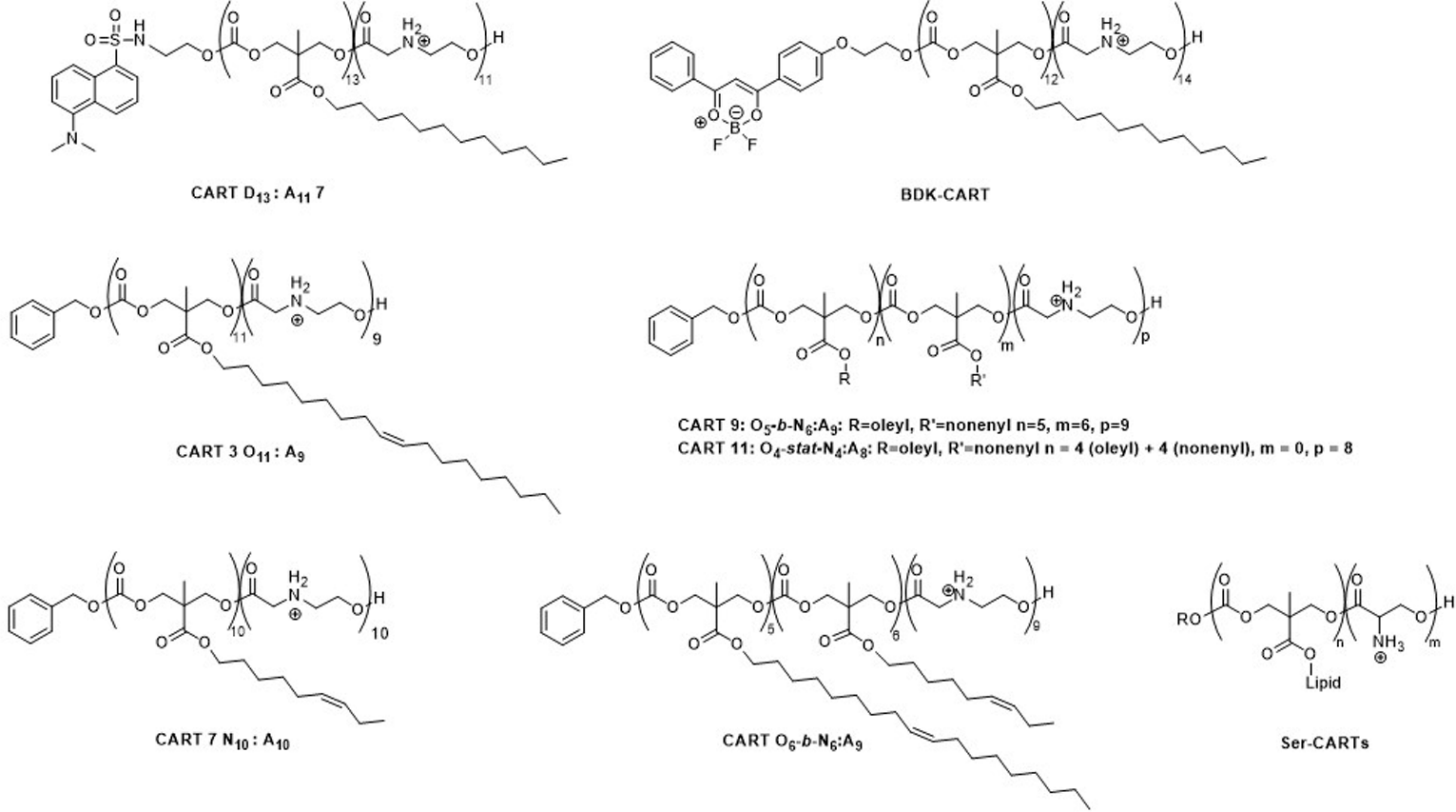
Representative chemical structure of CARTs

Among poly(amino ester) polymers, oligo(carbonate-*b*-α-amino ester) is one of the classical biodegradable charge-reversal examples, called charge-altering releasable transporters (CARTs) (Figure 7). The initial ammonium cations of CARTs could be partly deprotonated leading to an intramolecular cyclization, resulting in cationic initial ammonium to neutral amides transformation, ultimately promoting the release of loaded mRNA. In 2017, McKinlay et al. [40] firstly designed and synthesized unique oligomeric transporters for mRNA delivery using organocatalytic ROP (OROP) [40]. CARTs were employed to efficiently deliver antigen-coding mRNA into antigen-presenting cells (APCs), such as human peripheral blood mononuclear cells, finally achieve to activate the powerful antigen-specific immune response. Therefore, those CARTs successfully co-encapsulated with ovalbumin (OVA) mRNA and a Toll-like receptor ligand Cytosine-phosphate-Guanine (CpG) to simultaneously transfect and activate target cells to produce an immune response, which can eradicate large, established tumors *in vivo* [41]. Those studies highlight the potential of CARTs as simple, safe and effective vectors for mRNA vaccine delivery.

To fulfill the needs of lymphocyte transfection, McKinlay et al. [42] also constructed a combinatorial library of lipid-varied CARTs using two-step organocatalytic ROP. The oleyl CART 3 and nonenyl CART 7 could load the Luc mRNA to form stable complexes and deliver the mRNA into T and B lymphocytes (such as Jurkat cells). Delightfully, the hybrid-lipid CARTs 9 and 11 incorporating the same oleyl and nonenyl lipid show more effective transfection efficiency, even compared with the commercial agent Lipo2000. Such hybrid-lipid CARTs with high mRNA transfection efficiency could selectively deliver mRNA into CD4^+^ and CD8^+^ T cells of the spleen. These novel materials should be capable of new immunotherapy strategies and applications. This research provides design ideas a guide for establishing the new CARTs with lipid for delivery of mRNA to lymphocytes [42]. These functionalized biomaterials make possible new immunotherapy strategies and applications. Aiming against severe acute respiratory syndrome coronavirus-2 (SARS-CoV-2), they reported an alternative mRNA vaccine by using hybrid-lipid CARTs with co-encapsulating adjuvants CpG oligonucleotide (CpG-ODN) and receptor-binding domain (RBD)-specific encoding mRNA to exhibit strong and long-lasting T helper type 1 (TH1) T cell responses including CD4^+^ and CD8^+^ T cell memory [43]. They also established an alternative mRNA vaccine platform based on CARTs against the clinically relevant SARS-CoV-2 RBD antigen. The mRNA/CARTs vaccine co-formulated with adjuvants could promote the production of therapeutical specific neutralizing antibodies in the lung bronchial fluids of mice, and induce strong RBD-specific T cell responses. This mRNA/CARTs vaccine is the potent and flexible candidate as mRNA-based therapeutics against infectious diseases [43].

Furthermore, they synthesized the second generation CARTs based on oligo(serine esters), called as Ser-CARTs, which contained a charge-altering side chain amine and neutral serine-based byproducts upon degradation compared with first-generation CARTs. Ser-CARTs could be degraded into serine peptides at biological pH to enhance the mRNA release, resulting in efficient mRNA transfection and selective luciferase protein expression in the spleen. This tunable, targeting and efficient Ser-CARTs based mRNA delivery system could be explored for vaccination and immunotherapy [44].

**Figure 8.**
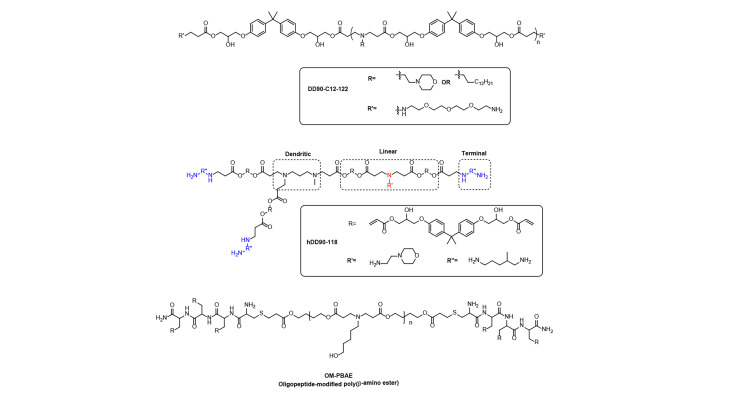
Representative chemical structure of PBAEs

PBAEs are another class of synthetic cationic biodegradable poly(amino ester) polymers, and their common synthesis method is to use diacrylates and primary or secondary amines by Michael addition reaction. According to the number of monomer amino groups and double bonds, PBAEs included linear, hyperbranched and crosslinked structures (Figure 8). PBAEs were firstly applied for DNA delivery in 2000 [45]. PBAEs as superior delivery candidates could stably encapsulate and release nucleic acids due to their reversible positive charge, high buffering capacity and rapid degradability under mild conditions [46], which could deliver different nucleic acids, including plasmid DNA, mRNA, siRNA, and circular dinucleotide (CDN), etc., into the target site [34, 47]. For examples, Kaczmarek et al. [48] synthesized a series of PBAEs terpolymers with alkyl tails and alkyl amines, and formulated them with polyethylene glycol (PEG)-lipid into nanoparticles for mRNA delivery. PEGylation could increase serum stability of PBAEs in the physiological environment, hence improving their mRNA delivery efficacy. The representative PEGylated PBAEs (DD90-C12-122) nanoparticles can successfully deliver luciferase mRNA to the lungs of mice [48].

In recently, Patel et al. [49] tailored hyperbranched PBAEs (hPBAEs) for functional *in vitro*-transcribed (IVT)-mRNA delivery to lung epithelium by nebulized mist for protein replacement therapy. The selected hyperbranched PBAE polymers (hDD90-118) could effectively encapsulate mRNA, promote cellular uptake and facilitate the cargo cytosolic release to undergo functional protein translation [49]. The results show that the hPBAE mRNA delivery system can further be employed for clinical pulmonary diseases treatment. The PBAE was further modified with the appropriate oligopeptide end-modified PBAEs (OM-PBAEs), which can specifically enrich in APC to achieve precision delivery in the spleen [50]. Furthermore, the potency and versatile OM-PBAEs may be appropriate for holding potential for immunotherapy.

## Conclusion and discussion

mRNA therapeutics hold great potential for biomedical applications, such as protein replacement therapy, vaccines for infectious diseases and cancer, genome editing, reprogramming of cell fates and so on. Biodegradable polyester materials with tunable structures and good safety profiles have been widely employed for mRNA delivery. Polyester materials could form the stable complex with mRNA via electrostatic interaction and were rapidly degraded under mild conditions to enhance the cargo release due to the ester-based backbone to improve transfection efficiency. Importantly, polyester materials with functional groups could specifically deliver the mRNA therapeutics into immune cells to induce the strong immune response.

Current progress suggested the feasibility of polyester materials for mRNA delivery, nevertheless, the development of polyester materials may be hampered by the following challenges for further clinical translation. Such as, their relative instability in the physiological environment reduces their *in vivo* delivery efficiency. And, the degradation mechanism of polyesters as mRNA delivery carriers is still unclear. In addition, the degraded monomers may cause potential toxicity and immunogenicity that should be taken into consideration. The long-term safety of these carriers must still be evaluated and the immune response to degradation products must be examined in detail.

Finally, according to the unique physicochemical properties of mRNA, the functionality of polyester materials must be further improved to meet the requirements for further application. Although there are current obstacles, with continuous efforts, scientists will develop novel polyester materials to be increasingly suitable for different nucleic acid therapeutics delivery.

## Abbreviations

aPACE: actuated poly(amine-co-ester)
CARTs: charge-altering releasable transporters
COVID-19: coronavirus disease 2019
mRNA: messenger RNA
PACE: poly(amine-co-ester)
PBAE: poly(β-amino ester)
PEG: polyethylene glycol
RBD: receptor-binding domain
ROP: ring-opening polymerization

## Declarations

### Copyright

© The Author(s) 2022.
